# Phase Engineering of Plasma‐Enhanced Atomic Layer Deposited Molybdenum Disulfide Using High Accuracy Direct Writing With Focused Li^+^ and Ga^+^ Ion Beams and Ultrashort Pulse Laser

**DOI:** 10.1002/smll.74834

**Published:** 2026-07-25

**Authors:** Marvin Krüner, Achim Nadzeyka, Malte Jonas Marius Julian Becher, Zhongquan Liao, Detlef Rogalla, André Clausner, Andreas Ostendorf, Michael Kahl, Claudia Bock

**Affiliations:** ^1^ Microsystems Technology Ruhr University Bochum Bochum Germany; ^2^ RAITH GmbH Dortmund Germany; ^3^ Applied Laser Technologies Ruhr University Bochum Bochum Germany; ^4^ Fraunhofer Institute for Ceramic Technologies and Systems IKTS Dresden Germany; ^5^ RUBION Ruhr University Bochum Bochum Germany

**Keywords:** atomic layer deposition, focused ion beam, intercalation, ${\mathrm{MoS}}_{2}$, phase engineering, ultrashort pulse laser

## Abstract

Two‐dimensional (2D) transition metal dichalcogenides (TMDCs), such as molybdenum disulfide (${\mathrm{MoS}}_{2}$), have significant potential as materials for the next generation of flexible electronics. They offer advantages over conventional semiconductors, particularly due to their tunable crystal phases. However, precise control of phase transformation in 2D layers deposited using low‐temperature processes in a bottom‐up approach for future electronics has not yet been mastered, which limits their integration into scalable device technology. This study demonstrates the control of the phase composition of plasma enhanced atomic layer deposited (PEALD) ${\mathrm{MoS}}_{2}$ films by direct writing with focused ${\mathrm{Li}}^{+}$ and ${\mathrm{Ga}}^{+}$ ion beams, and retransformation into the 2H phase by ultrashort pulse laser processing. The ion beam‐treated films exhibit excellent long‐term stability and are therefore suitable for industrial processes.

## Graphical abstract

In the context of phase heterostructures on two‐dimensional materials, a high‐precision direct writing approach is employed. This involves using a focused ion beam to transform molybdenum disulfide to the metallic 1T phase, followed by retransformation via ultrashort pulse laser processing. This mask‐free, wafer‐compatible technique enables phase control to be used as a design parameter for next‐generation electronic and optoelectronic systems.

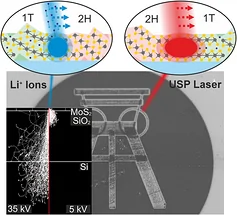

## 1 Introduction

**FIGURE 1:**
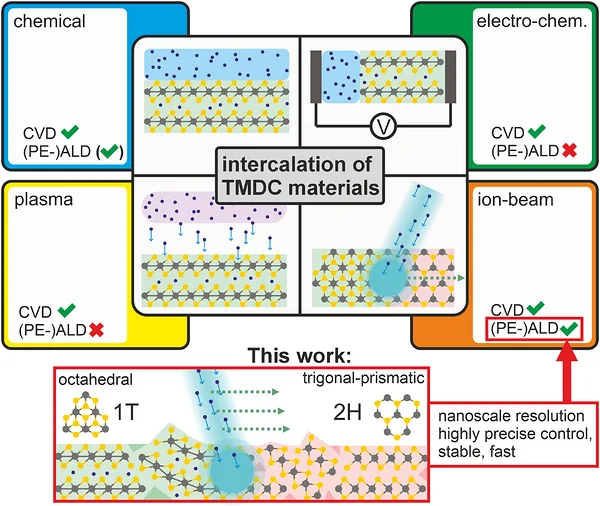
Schematic drawing of methods for phase transformation in ${\mathrm{MoS}}_{2}$ by intercalation on ideal monocrystalline films with information which processes were already used on CVD or (PE‐)ALD films. top left: liquid‐phase chemical intercalation, bottom left: vapor‐phase chemical intercalation, top right: electro‐chemical intercalation, bottom right: ion beam intercalation and below: ion beam intercalation on polycrystalline PEALD films (this work).

Due to its unique properties as a two‐dimensional $\left(\text{2D}\right)$ material, molybdenum disulfide (${\mathrm{MoS}}_{2}$) has emerged as a promising material for future electronics technologies. In its atomically thin form, it consists of a layer of molybdenum sandwiched between two layers of sulfur. ${\mathrm{MoS}}_{2}$ combines a considerable bandgap, which can be adjusted via the layer thickness, with excellent electrostatic control, mechanical flexibility, and chemical stability. This makes it a strong candidate for next‐generation transistors, (flexible) electronics and optoelectronic devices [1, 2, 3]. Unlike graphene, ${\mathrm{MoS}}_{2}$‐based devices can achieve high on/off current ratios of up to 10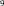 due to their intrinsic bandgap, which is crucial for low‐power electronic applications with mobilities of up to 700 ${\mathrm{cm}}^{2}{\;(\mathrm{Vs})}^{-1}$ [4, 5]. In addition to its use in conventional electronics, ${\mathrm{MoS}}_{2}$ is highly relevant for More‐than‐Moore concepts, particularly neuromorphic electronics, quantum electronics, including valleytronics, quantum dots, single‐photon emitters and quantum detectors [6, 7, 8, 9, 10]. ${\mathrm{MoS}}_{2}$ is a promising material for electrocatalysis, particularly for the hydrogen evolution reaction (HER) [11]. Thanks to the polymorphism in ${\mathrm{MoS}}_{2}$, these applications are becoming more powerful and innovative. Different crystal phases, most notably the semiconducting 2H phase and the metallic or semi‐metallic 1T and 1T' phases, exhibit fundamentally distinct electrical, chemical, and mechanical properties despite their identical chemical composition. Phase engineering in two‐dimensional materials has emerged as a powerful concept to overcome the intrinsic limitations of conventional semiconductor scaling and enable novel device functionalities beyond the CMOS paradigm [12, 13]. By locally switching between semiconducting and metallic states, ${\mathrm{MoS}}_{2}$ enables devices whose functionality is defined by phase rather than by conventional doping or complex material stacks. Unlike silicon technology, which is approaching its physical and architectural limits, this phase‐controlled approach provides unparalleled flexibility, which offers significant advantages in terms of low‐resistance contacts, reconfigurable device architecture and monolithic function integration in electromagnetical devices. The selective synthesis of these crystal phases is an active area of research, but phase transformations of ${\mathrm{MoS}}_{2}$ also happen post deposition under the influence of voltage, defects, temperature, electric fields, alloys, or ion intercalation [12, 14, 15]. An effective and widely studied route for phase transformations in ${\mathrm{MoS}}_{2}$ is based on ion intercalation which means inserting atoms, ions or molecules between the layers of layered materials. Intercalation with alkali metals is particularly promising, as theoretical calculations have shown that it can stabilize the 1T phase of ${\mathrm{MoS}}_{2}$ compared to the 2H phase [16]. There are several strategies for achieving phase transformation by intercalation, which are schematically depicted in Figure 1. Chemical intercalation, most notably using alkali metals such as lithium, enables the insertion of ions and electrons into the van der Waals gaps of 2H‐${\mathrm{MoS}}_{2}$. The donation of electrons by intercalated lithium atoms results in the destabilization of trigonal prismatic coordination and a transition toward the octahedrally coordinated 1T or distorted 1T' phases [15, 17]. Throughout the manuscript, we use the umbrella terms “1T ${\mathrm{MoS}}_{2}$” or “1T phase” to refer collectively to the octahedrally coordinated 1T/1T' variants, as our measurements do not allow an unambiguous distinction between them in our samples. Chemical intercalation is driven by concentration gradients and can be carried out in a solution containing the intercalant or in the liquid phase of the intercalant over a period ranging from hours to days. Another technological option is to evaporate the intercalant in a container with the 2D material. This method requires high temperatures but drastically reduces process times [15]. Beyond chemical approaches, electrochemical intercalation provides enhanced control over the phase transformation process, allowing reversible and spatially selective phase switching with ionic liquid electrolytes through applied gate voltages [15]. Electrochemically driven intercalation enables dynamic tuning of phase composition and has been exploited in reconfigurable electronics, memristive devices and neuromorphic systems, where nonvolatile or analog conductance states can be realized [15]. Importantly, intercalation‐induced phase transformations often result in phase coexistence and metastable domain structures, giving rise to phase boundaries that exhibit distinct electronic and catalytic properties [18]. Another approach to achieve partial phase transition in $\mathrm{Mo}S_{2}$ is Argon and $\mathrm{Ar}/O_{2}$ plasma treatment [19, 20]. Finally, to the best of our knowledge, there is only one paper discussing the phase transformation of 2D films using FIB. Xiao et al. successfully used a gallium FIB with high acceleration voltages to transform monocrystalline ${\mathrm{MoTe}}_{2}$ sheets into the 1T phase [21].

So far, most intercalation methods have only been tested on chemically vapor deposited (CVD) or exfoliated monolayers, or on systems comprising a few layers. PEALD ${\mathrm{MoS}}_{2}$ films contain a much higher density of grain boundaries than high‐temperature CVD‐grown 2D layers. Defects have been shown to strengthen the bonding of lithium within ${\mathrm{MoS}}_{2}$, enhancing lithium adsorption [22]. At the same time, grain boundaries, depending on their atomic structure and chemistry, can impede lithium‐ion diffusion in TMDCs [23]. This complicates the direct transfer of CVD‐proven techniques to PEALD films because lithium transport pathways and kinetics are fundamentally different. However, reduced lithium mobility is not necessarily detrimental. By limiting post‐intercalation lithium diffusion, grain boundaries can help retain lithium locally, which can pin phase fronts and make lithium‐induced phase heterostructures more spatially defined and potentially more stable over time. To our knowledge, successful phase transformation and retransformation has been achieved in polycrystalline ALD‐deposited ${\mathrm{MoS}}_{2}$ layers once. Becher et al. induced a 2H‐to‐1T phase transformation in PEALD ${\mathrm{MoS}}_{2}$ films by immersing them in a butyllithium solution for 72 h. Retransformation to the 2H phase was then achieved using a USP laser process [24]. However, this procedure is time‐consuming, difficult to control in terms of alkali metal content, requires extensive yet ineffective cleaning processes so that lithium residues remain on the surface and poses a high risk of delamination of the 2D film.

Using a focused ion beam can provide a decisive boost to innovation. FIB processing of thin films modifies surface structure and properties through mechanisms such as ion implantation, ion milling, ion‐beam‐induced etching, deposition [25]. It offers a powerful platform for maskless, resist‐free direct writing of nanoscale structures with exceptional precision in terms of positioning and ion dose [26, 27]. Apart from the work of Xiao et al. [21] demonstrating phase transition on ${\mathrm{MoTe}}_{2}$, gallium ion beams have primarily been used in TMDC applications for ablation, sheet modification, thinning, and patterning [28, 29]. Additionally, helium ion beams are employed for non‐destructive imaging applications [30] and preferential sputtering of sulfur from ${\mathrm{MoS}}_{2}$ thin films, which leads to metallic behavior [31, 32]. The liquid metal alloy ion source (LMAIS) became a well‐established FIB source technology providing a versatile approach for delivering various ion species from a single source. Despite the potential relevance of LMAIS in the context of phase transitions in 2D materials, where they can provide the alkali metals necessary for improving the long‐term stability of the metallic phase, it has not yet been used alongside advanced 2D technology. Combining alkali‐metal‐based phase engineering with FIB‐enabled patterning paves the way for deterministic, reconfigurable phase control in two‐dimensional materials.

In this work, we present the first phase engineering of PEALD‐grown ${\mathrm{MoS}}_{2}$ using focused ion beam direct writing with an innovative multi‐species ion source consisting of gallium, lithium and bismuth, and subsequent ultrashort pulse laser retransformation to the semiconducting phase [33]. Gallium and bismuth are suitable for ion beam milling due to their high mass. Using Li ions in their stead offers two key advantages. First, the lower mass of the ions enables a wider range of parameters prior to amorphization, in which the phase transformation can occur, providing ultimate control over the transformation. Second, lithium is the lightest alkali metal and significantly improves the long‐term stability of the metallic phase of ${\mathrm{MoS}}_{2}$ through intercalation. This added value is crucial for future industrial applications. This method tackles the negative aspects of the wet chemical approach by reducing three steps into one using focused ion beams to directly write the metallic phase into ${\mathrm{MoS}}_{2}$ sheets. It reduces processing times from over three days [24] to a few minutes or hours on a wafer scale, enables highly precise control of the lithium amount in the 2D‐film and eliminates the undesirable wet chemical steps and thus the risk of delamination. Last but not least, an ultrahigh‐resolution (around 2.9 nm for a 35 keV ${\mathrm{Li}}^{+}$‐ion beam) phase control can be achieved for future ultrascaled devices [34].

## 2 Results and Discussion

Within this study, gallium (Ga) and lithium (Li) ion beams are used. Gallium is known as the standard ion species for FIB processes and provides a good basis for comparing the results with those of other groups. Lithium is the lightest metal in the periodic table, suitable for application in LMAIS and is a highly promising material for alkali metal intercalation‐induced phase transitions in TMDC materials. With the help of the GaBiLi LMAIS [33], it is possible to compare the effects of the two ion species on PEALD‐grown ${\mathrm{MoS}}_{2}$ layers on the same film and in the same machine by simply switching the ion beam. At the same time, the influence of ion mass on phase transformation can be investigated. As expected and already demonstrated with graphene, heavier ions cause greater damage and higher defect densities than lighter ions. This significantly impacts the structural integrity and performance of 2D materials, highlighting the importance of conducting studies on ion irradiation as a function of both ion mass and dose for effective material development. After reviewing several studies most of them are done for doses between $10^{12}$ and $10^{15}\;{\text{cm}}^{-2}$ [29, 35, 36, 37, 38] with only a few studies starting at doses below $10^{11}\;{\text{cm}}^{-2}$ [39]. Above $10^{16}{\text{cm}}^{-2}$ most ion beam applications on 2D materials revolve around patterning and milling even with low mass ${\mathrm{He}}^{+}$ ion beams [31, 40]. Thus, we fixed our parameter range for the exposure of a 10 nm thick PEALD‐grown ${\mathrm{MoS}}_{2}$ film to doses of $10^{13}{\text{cm}}^{-2}$ $\leq D_{n}\leq$ $10^{16}{\text{cm}}^{-2}$ at the same acceleration voltage (Figure S1). Because the probability of damage to 2D materials is lower at low energy, we selected an AV of 5 kV, the lowest available at the FIB system used for this study, and a standard AV of 35 kV for comparison. Details on the selected sizes of the writing fields and parameters can be found in Section S1. After irradiation with the ion beam, optical micrographs reveal a darkening of the color with increasing doses. This may be a result of a reduction in layer thickness caused by ablation, which is more likely to occur with the heavier ${\mathrm{Ga}}^{+}$ ions. Alternatively, it may be due to the altered optical properties resulting from the 1T transformation caused by Li intercalation [41].

**FIGURE 2:**
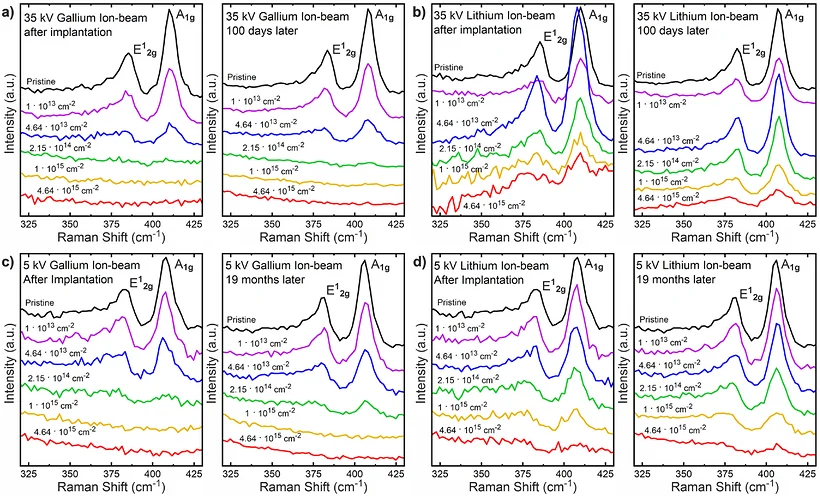
Four pairs of waterfall Raman spectra of ion beam irradiated ${\mathrm{MoS}}_{2}$. Each pair depicts a different ion dose and acceleration voltage combination directly after implantation and 100 days/19 months later. (a) 35 kV gallium ion‐beam. (b) 35 kV lithium ion‐beam. (c) 5 kV gallium ion‐beam. (d) 5 kV lithium ion‐beam

**FIGURE 3:**
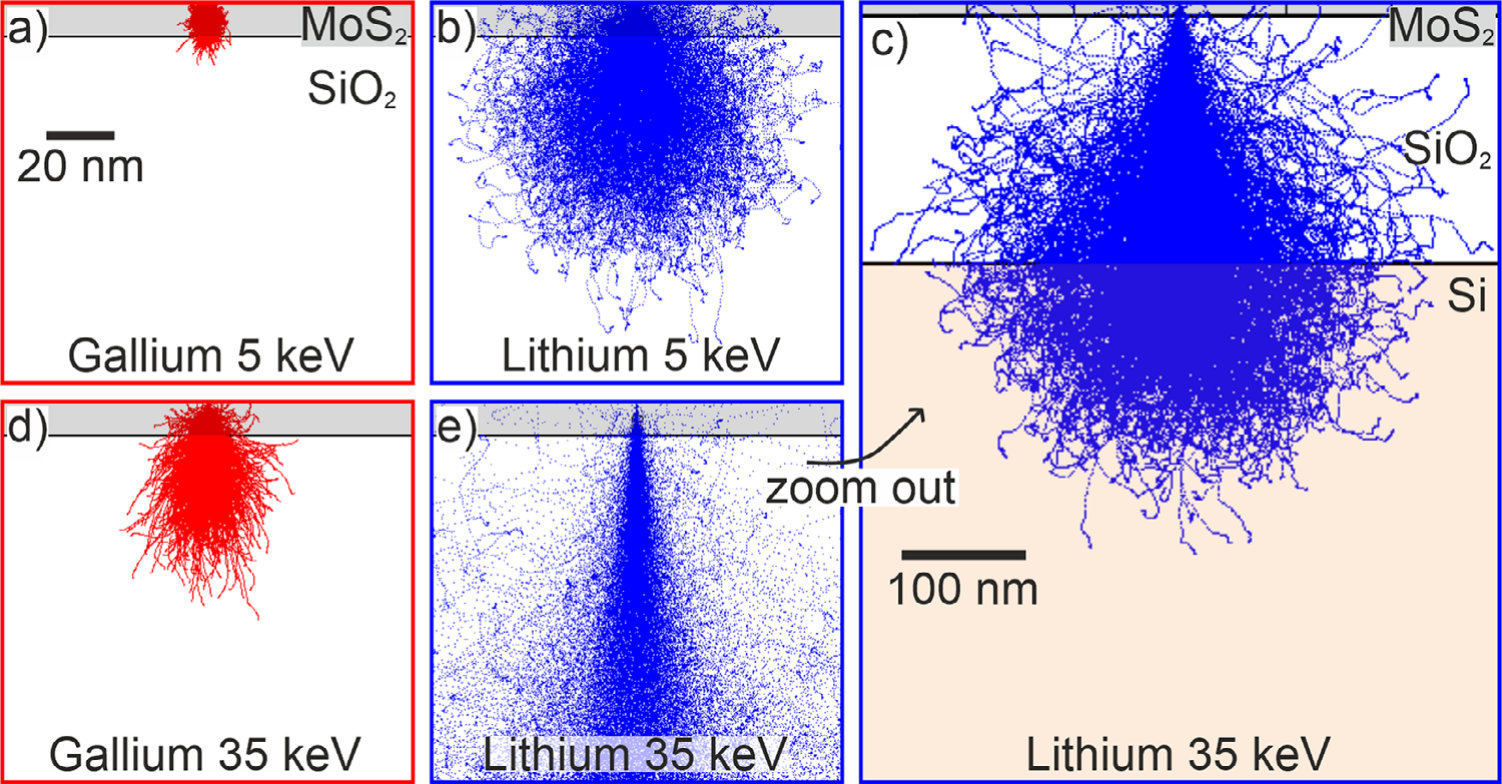
TRIM simulations of different ion species from a GaBiLi LMAIS at 35 and 5 keV beam energy penetrating in a layer structure consisting of 10 nm ${\mathrm{MoS}}_{2}$ on 200 nm ${\mathrm{SiO}}_{2}$ on silicon: (a) ${\mathrm{Ga}}^{+}\mathrm{ion}$ beam, 5 keV (b) ${\mathrm{Li}}^{+}$ ion beam, 5 keV, (d) ${\mathrm{Ga}}^{+}$ ion beam, 35 keV (e) ${\mathrm{Li}}^{+}$ ion beam, 35 keV and (c) ${\mathrm{Li}}^{+}$ ion beam, 35 keV at a larger scale

Figure 2 shows paired Raman spectra of writing fields with different doses of Li and Ga ions at an AV of 5 kV (bottom row) and 35 kV (top row), taken immediately after implantation (left) and 100 days/19 months later (right). All graphs display the general trend that the characteristic Raman peaks of the 2H phase decrease with increasing dosage. Gallium‐irradiated ${\mathrm{MoS}}_{2}$ loses all Raman peaks at a dose of $2.15\times 10^{14}{\text{cm}}^{-2}$, and no significant difference can be seen in the Raman spectra at acceleration voltages of 5 kV and 35 kV. At the same time, the intensity ratio of the LA(M) peak to the $A_{1g}$ peak increases from an initial value of 0.2 to 0.65 for an AV of 5 kV, to 0.8 for an AV of 35 kV at a dose of $10^{14}{\text{cm}}^{-2}$. This indicates the rapid amorphization of the film. Lithium irradiation exhibits different behavior, with significant differences between the two acceleration voltages. At 5 kV, the intensities of the Raman peaks gradually decrease, only disappearing at around $4.64\times 10^{15}{\text{cm}}^{-2}$. In contrast, at 35 kV, the peaks never completely disappear, even at the highest doses. The intensity ratio of the LA(M) peak to the $A_{1g}$ peak increases more slowly in comparison to the Ga ion beam. It is 0.3 at an accelerating voltage (AV) of 5 kV and 0.65 at an AV of 35 kV for a dose of $1\times 10^{14}{\text{cm}}^{-2}$. The heavy gallium ions are characterized by a higher sputter rate and a lower penetration depth than Li ions [33]. While elastic collision with the substrate dominates the interaction for gallium ion beams at both 35 and 5 keV ion energies, inelastic collisions are more pronounced at higher ion energies for lighter Li ions, elastic collisions only take place after some energy has been lost in the uppermost nanometers of the material. Qianhuang Chen et al. demonstrated this effect when irradiating silicon with a helium beam at comparable acceleration voltages [42]. In contrast to the nuclear stopping, the electronic stopping mechanism does not directly displace substrate atoms, meaning higher dosages are required to turn the ${\mathrm{MoS}}_{2}$ film amorphous. This is expected, since the heavy gallium ions mostly amorphize the layer structure, and the energy is mainly deposited in the uppermost nanometers of the sample, even at 35 kV AV. The Raman intensity rises when using a lithium‐ion beam accelerated to 35 kV and a dose of $4.64\times 10^{14}\mathrm{cm}$ 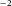. This increase in intensity can still be observed after 19 months, indicating successful tempering of the ${\mathrm{MoS}}_{2}$ at these values. Since electronic deceleration involves large amounts of energy but only minor disorder in a small spot size, local heating can occur, causing an annealing effect in the irradiated ${\mathrm{MoS}}_{2}$ film. In monocrystalline ${\mathrm{MoS}}_{2}$ the $J_{1}$, $J_{2}$ and $J_{3}$ peaks are typically associated with the 1T/1T' phases. These are very weak compared to the $E_{2g}^{1}$ and $A_{1g}$ peaks and have not yet been observed in polycrystalline ${\mathrm{MoS}}_{2}$ grown by PEALD. The Raman peaks of this material are increasingly faint due to their low intensity and can therefore be lost in the background signal. Additionally, the $J_{3}$ peak is located close to the $E_{2g}^{1}$ mode and the silicon peak, which may obscure it. The most intense J peak is the $J_{2}$ peak, which occurs at 222 ${\mathrm{cm}}^{-1}$ and is therefore close to the broad LA(M) peak at 227 ${\mathrm{cm}}^{-1}$ induced by disorder. In a recent publication, Maddouri et al. showed that the $J_{1}$ peak, often cited as the sole evidence of the 1T/1T' phase in many studies, may also be a characteristic of ${\mathrm{MoO}}_{3}$. This is because 2D films often oxidize when exposed to high levels of Raman laser power [43]. To mitigate this, we employed low Raman intensities and extended integration times during spectral acquisition. A Raman spectrum recorded under these conditions and covering the relevant wavenumber range can be found in Section S2, where it is discussed in more detail. As expected for films deposited by PEALD, no clear signatures of the J peaks were found, as has been the case elsewhere. However, the slight shift of the broad peak at around 225 ${\mathrm{cm}}^{-1}$ toward lower wavenumbers in the Li‐intercalated film can be interpreted as an indication of an overlapping of the disorder induced LA(M) and emerging $J_{2}$ peak indicating successful phase transition. Raman spectra of samples irradiated with a 35 keV lithium beam also show a significant deterioration of the silicon peak at 521 ${\mathrm{cm}}^{-1}$ with increasing dose (Figure S3). This is due to the light ions having a high penetration depth, which exceeds the 200 nm thickness of the ${\mathrm{SiO}}_{2}$ layer. To estimate implantation depths of lithium and gallium ion beams at 5 and 35 kV acceleration voltage, we conducted TRIM‐Simulations of a point‐shaped ion beam (Figure 3) [44]. The layer structure of 10 nm ${\mathrm{MoS}}_{2}$ on 200 nm ${\mathrm{SiO}}_{2}$ on silicon was used assuming perfect stoichiometry and a bulk density of 5.06 g ${\mathrm{cm}}^{-3}$ for ${\mathrm{MoS}}_{2}$. At a voltage of 35 kV, the light lithium ions penetrate the entire ${\mathrm{MoS}}_{2}$/${\mathrm{SiO}}_{2}$ stack and amorphize the underlying crystalline silicon. The heavier Ga ions penetrate to a depth of 40 nm into the layer stack. This result is consistent with the Raman spectra of the layers treated with Ga ions. None of them show any change in the Si peaks. Using an acceleration voltage of 5 kV stops all the Ga ions in the upper ${\mathrm{MoS}}_{2}$ layer, while many of the lighter Li ions penetrate the ${\mathrm{SiO}}_{2}$ layer.

In a subsequent step, the influence of the FIB process on the stoichiometry of the ${\mathrm{MoS}}_{2}$ film was investigated using Rutherford backscattering measurements. For this, circles with a diameter of 750 μm were written by a Ga‐ion and Li‐ion beam at an AV of 5 kV with ion doses approximately corresponding to the ion doses at which Raman signals disappear in previous tests: $D_{Li}$ = $3\times 10^{15}$ ${\mathrm{cm}}^{-2}$ and $D_{Ga}$ = $2.15\times 10^{14}$ ${\mathrm{cm}}^{-2}$. As expected, the initial film is sulfur‐rich, with an S/Mo ratio of 2.11. The sulfur excess is slightly reduced by lithium‐ion beam irradiation to 2.07, and despite the lower dose, this value is reduced further by gallium‐ion beam treatment (S/Mo ratio: 2.04). The preferential sputtering of sulfur in ion beams is consistent with the experimental and theoretical results of other research groups and is due to the lower binding energy of sulfur compared to molybdenum [28]. The more pronounced effect of gallium on the ${\mathrm{MoS}}_{2}$ stoichiometry originates from the different sputter yield of the ion species. Schmidt et al. report sputter yields of 1.80, 0.29, and 0.05 for gallium, beryllium and helium respectively, the next heavier and lighter ions to lithium, at acceleration voltages of 12.5 kV [45]. The disappearance of the $E_{2g}^{1}$ and $A_{1g}$ peaks in the Raman spectrum during Li‐ion treatment ($D_{Li}$ = $4.64\times 10^{15}$ ${\mathrm{cm}}^{-2}$), while maintaining the layer structure and nearly ideal S/Mo stoichiometry of 2.07, are strong indications of a successful phase transformation from the 2H phase to the 1T phase. This transformation was still stable 19 months after Li intercalation (Figure 2). Optical images and Raman spectra of these implantations can be found in Figure S4.

**FIGURE 4:**
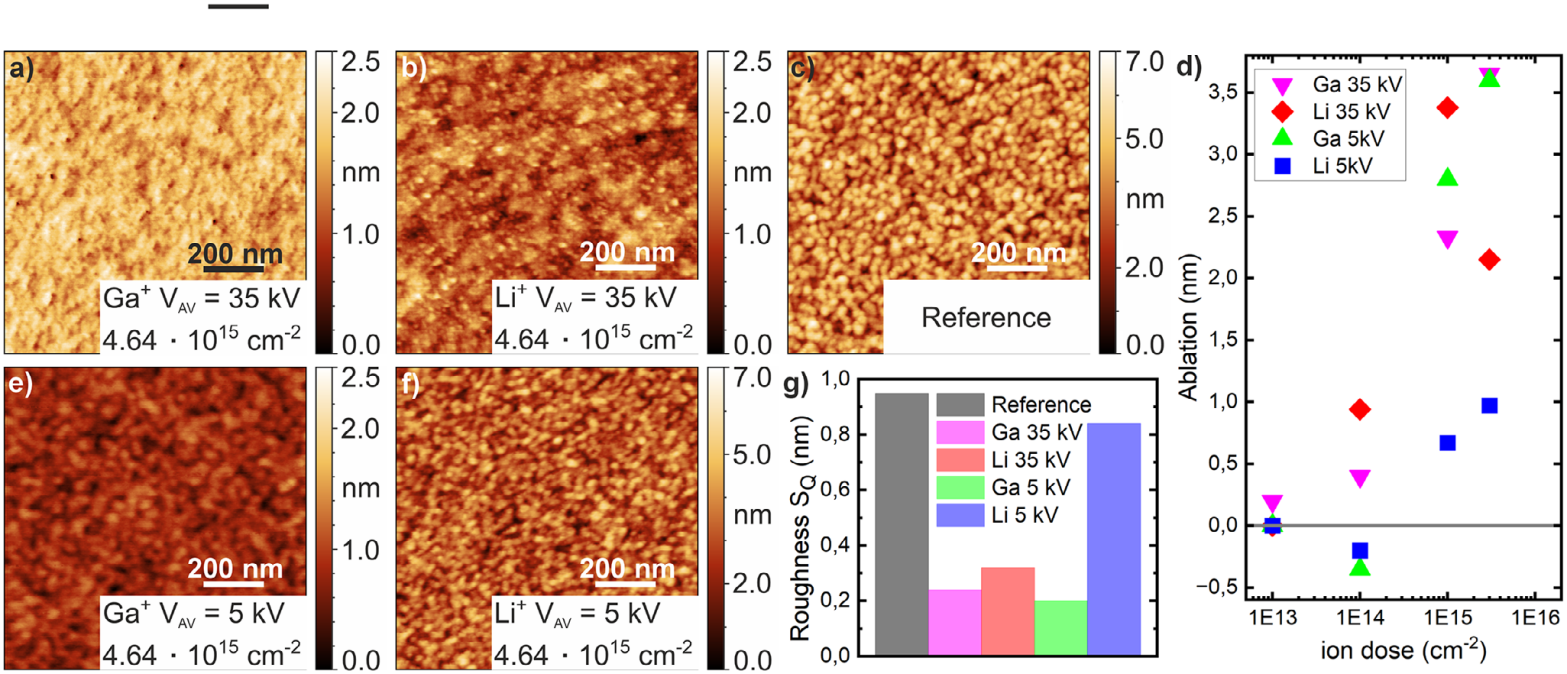
Atomic force micrographs (1 μm x 1 μm) of ${\mathrm{MoS}}_{2}$ films with implantations of gallium (a), (e) or lithium (b), (f) ion doses of $4.64\times 10^{15}$ ${\mathrm{cm}}^{-2}$ to at acceleration voltages of 5 kV (e), (f) or 35 kV (a), (b). (c) AFM image of the untreated films. The root mean square roughnesses are given in (g) and (d) depicts differences in ${\mathrm{MoS}}_{2}$ sheet thickness over ion dose of all four parameter combinations measured with AFM.

**FIGURE 5:**
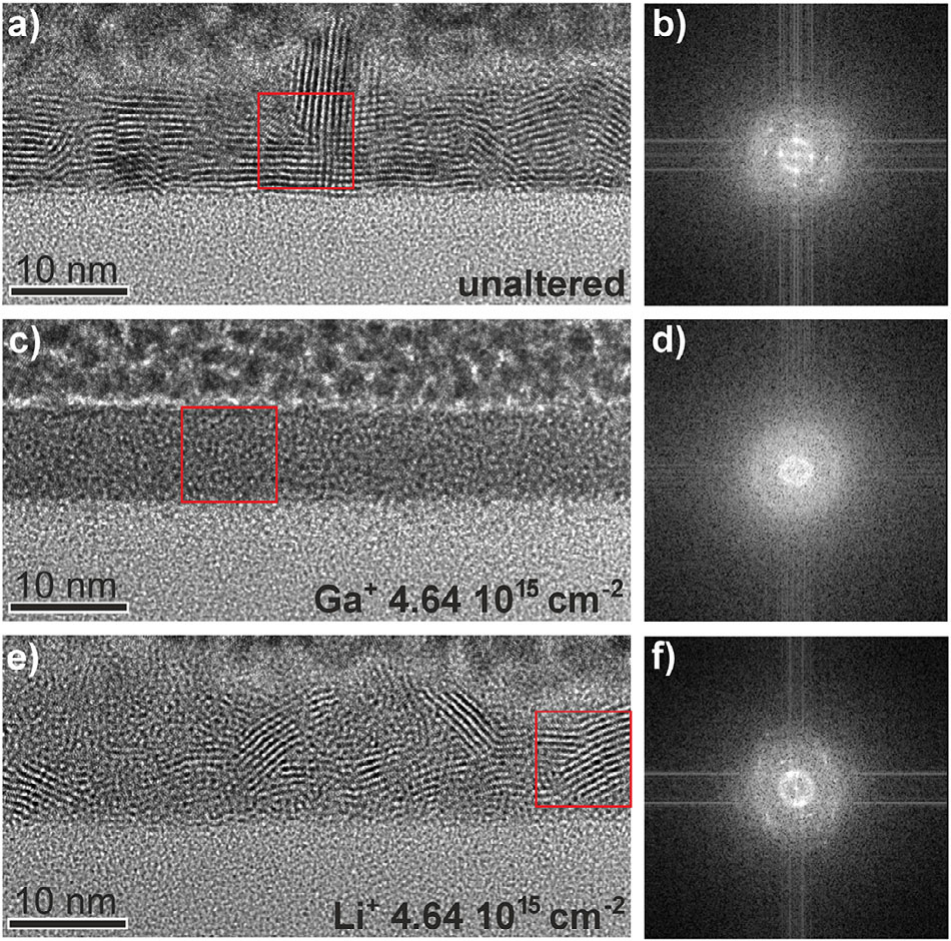
HRTEM Cross‐section Images of one unaltered sample as well as lithium and gallium dosages of $4.64\times 10^{15}$ ${\mathrm{cm}}^{-2}$ (AV = 5 kV) with cropped FFT images of 8 nm × 8 nm areas inside the ${\mathrm{MoS}}_{2}$ layer. A carbon capping layer deposited before TEM imaging is above the ${\mathrm{MoS}}_{2}$ thin film and substrate ${\mathrm{SiO}}_{2}$ below. (a, b) unirradiated reference layer. (c, d) irradiated with a ${\mathrm{Ga}}^{+}$ dose of $4.64\times 10^{15}$ ${\mathrm{cm}}^{-2}$. (e, f) treated with a $4.64\times 10^{15}$ ${\mathrm{cm}}^{-2}$ ${\mathrm{Li}}^{+}$ ion dose.

Based on Raman analysis, the disappearance of $E_{2g1}$ and $A_{1g}$ signals in the Raman spectra at $D_{Ga}=2.15\times 10^{14}\;\mathrm{cm}$ 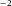 for ${\mathrm{Ga}}^{+}$‐ions and $D_{Li}=4.4\times 10^{15}\;\mathrm{cm}$ 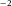 for ${\mathrm{Li}}^{+}$ ions can be observed. However, it is difficult to determine whether this is due to complete removal, amorphization, or a phase transformation. Therefore, a comprehensive AFM analysis was conducted. To reliably measure even the smallest removal and the impact of ion beam treatment on the surface roughness, a grid structure with ${\mathrm{Ga}}^{+}$ and ${\mathrm{Li}}^{+}$ ion doses ranging from $10^{13}$ to $10^{16}$ ${\mathrm{cm}}^{-2}$ was written at acceleration voltages of 5 and 35 kV, respectively. Optical micrographs of this can be found in Figure S5. The topography of the ${\mathrm{MoS}}_{2}$‐film changes drastically for Ga ion treated films (Figure 4a,e), when compared to the untreated ${\mathrm{MoS}}_{2}$ film (Figure 4c). The ablation threshold for heavy Ga ions at an acceleration voltage of 35 kV is around $10^{13}$ ${\mathrm{cm}}^{-2}$, whereas for lighter Li ions it is closer to $10^{15}$ ${\mathrm{cm}}^{-2}$. Higher acceleration voltages result in lower ablation thresholds allowing faster ion beam milling process. The AFM images also show that the topography of the ${\mathrm{MoS}}_{2}$ films treated with a Li ion beam at 5 kV and with a dose of $4.64\times 10^{15}$ ${\mathrm{cm}}^{-2}$ (Figure 4f) is similar to that of the untreated film (Figure 4c), suggesting that the film has sustained minimal damage. When the 2D film is irradiated with lithium ions accelerated at 5 kV, the resulting topography and roughness are very similar to that of the initial layer (Figure 4g). Ablation of the ${\mathrm{MoS}}_{2}$ film is very high at high gallium ion doses and acceleration voltages (Figure 4d). At the highest doses, the layer thickness is almost halved. A lithium‐ion beam accelerated to 5 kV causes only negligible ablation of the ${\mathrm{MoS}}_{2}$ film at doses of up to $1\times 10^{14}$ ${\mathrm{cm}}^{-2}$, and even at the highest dose, the ablation is less than one nm. When using a Li beam, elastic scattering processes only appear to gain the upper hand over inelastic ones at doses above $1\times 10^{15}$ ${\mathrm{cm}}^{-2}$ and an AV of 5 kV. Since ${\mathrm{Ga}}^{+}$ ions at 35 keV do not fully remove the 2D material although the 2H phase Raman signal disappears completely, it can be assumed that this is due to the film becoming amorphous. This also aligns with the AFM images, which depict the films as having very low roughness. Consequently, in combination with the detrimental effect of device functionalities due to deep penetration (Figure 3), the following sections focus on acceleration voltages of 5 kV. Figure 5 shows cross‐sectional high‐resolution transmission electron microscopy (HR‐TEM) measurements as well as a cropped fast Fourier transformation (FFT) plot from inside the ${\mathrm{MoS}}_{2}$ layer. This analysis was done on ${\mathrm{MoS}}_{2}$ layers after 5 keV ion irradiation with a dose of $4.64\times 10^{15}$ ${\mathrm{cm}}^{-2}$ with both ion species and on a reference sample without FIB treatment. As expected for PEALD‐deposited ${\mathrm{MoS}}_{2}$, the initial film reveals a clear polycrystalline structure with most of the layering in the lateral orientation, but also some crystallites oriented out of plane. High doses of gallium completely amorphized the molybdenum disulfide layer, which is consistent with the disappearance of the layered structure, the diffuse FFT image and the vanished $E_{2g}^{1}$ and $A_{1g}$ peaks of the 2H‐phase in the Raman spectra. ${\mathrm{Ga}}^{+}$ focused‐ion‐beam exposure deposits energy through the nuclear stopping mechanism, knocking atoms from lattice sites, preferentially sputtering chalcogen species, and implanting Ga into the near‐surface region [46, 47]. This process disrupts long‐range order, alters stoichiometry, and promotes bond rearrangements, resulting in an amorphous layer. When a ${\mathrm{Li}}^{+}$ beam is used instead of the ${\mathrm{Ga}}^{+}$ beam, layered structures are still observed, while the Raman peaks were not visible at this dose (Figure 2d). Also some weak periodic point reflections can be seen in the FFT. This emphasizes a successful phase transformation from 2H to 1T is taking place. The interlayer distance was determined by evaluating histogram and FFT data. In both pristine and lithium irradiated layers values between 0.6 and 0.65 nm were determined, comparable to literature values of 0.62 nm for 2H and ranging from around 0.65 nm to over 1 nm for 1T ${\mathrm{MoS}}_{2}$ prepared by n‐butyllithium intercalation [48, 49, 50, 51]. The TEM images confirm that the thickness of the ${\mathrm{MoS}}_{2}$ layer under the Ga beam decreases significantly, to approximately 8 nm, while the thickness of the sample treated with the ${\mathrm{Li}}^{+}$ beam remains nearly unchanged. With the lithium‐ion dose selected here, it is noticeable that the crystallites are smaller than in the untreated sample. In the upper part of the layer, some regions are amorphous. The degree of crystallinity should be directly reflected in the surface roughness of the samples, which is why it was determined using atomic force microscopy. The RMS roughness amounts 1.0 nm for the initial film, 0.2 nm for the Ga‐ion irradiated films, 0.3 and 0.8 for the Li irradiated films with 35 and 5 kV acceleration voltage respectively (Figure 4). A closer look at the interface in the TEM images reveals a deterioration of the originally sharp interface between ${\mathrm{MoS}}_{2}$ and ${\mathrm{SiO}}_{2}$, which is less pronounced when using the Li ion beam than when using the Ga ion beam. Clearly, even when using very light ions, the lowest acceleration voltage available in the Velion system is still too high to rule out damage to the interfaces and partial amorphization. At the current stage of plant technology it is not possible to distinguish a specific critical dose for phase transition and amorphization from lithium FIB exposure. In the long term, plant technology will also have to be adapted for future 2D electronics to prevent even the slightest damage to the 2D films, the dielectric substrates or their interfaces.

**FIGURE 6:**
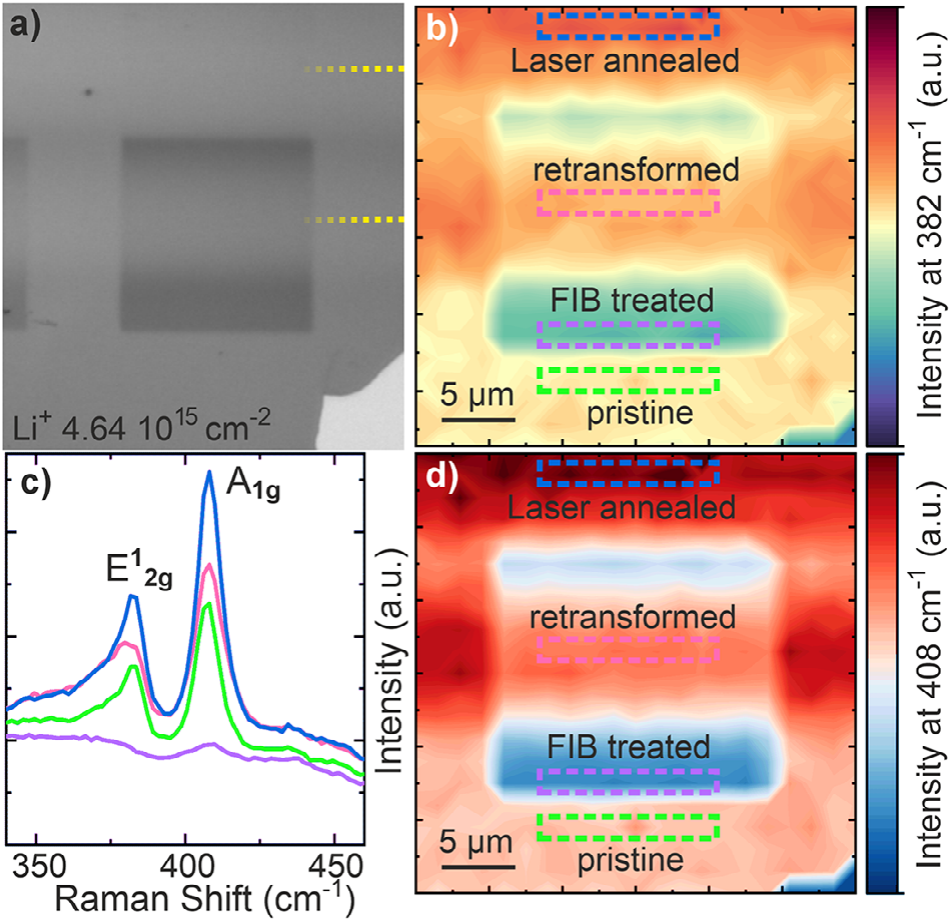
(a) Blue light optical microscopy of an irradiated square with a lithium ion dose of $4.64\times 10^{15}$ ${\mathrm{cm}}^{-2}$. Dotted yellow lines indicate the middle of the USP laser path. (b), (d) Mappings of the $E_{2g}^{1}$ and $A_{1g}$‐peak Raman intensities of two irradiated squares and (c) Raman spectra corresponding to the areas marked by dotted rectangles in the same color (right column). The spectra show mean values of eight measurement points in each area (FIB‐Irradiated (purple), FIB‐Irradiated and laser‐retransformed (pink), Laser annealed (blue) and pristine (green).

Having successfully transformed the material with high local resolution using lithium ions, the next step is to convert the 1T phase back to the 2H phase using ultrashort pulse laser processing (USP). By introducing energy in periods shorter than thermal diffusion, USP excitation enables non‐equilibrium phase control while minimizing substrate damage, making it suitable for flexible substrates as well. Based on previous experiments with ultrashort pulse lasers on the phase transition of 2D ${\mathrm{MoS}}_{2}$ [24], the with Li ion beam irradiated writing fields were scanned by a laser beam passing from left to right. Figure 6 depicts optical micrographs of implanted squares with a crossing laser path (lighter stripe, Figure 6a), mappings of the Raman $E_{2g}^{1}$ and $A_{1g}$ peak intensity mapping of the 2H phase across each square (Figure 6b,d) and Raman spectra corresponding to the relevant areas marked in the right column inside and outside of implanted regions. The characteristic $E_{2g}^{1}$ and $A_{1g}$ Peak of the initial ${\mathrm{MoS}}_{2}$ film (green graph) vanish by FIB laser treatment (purple graph) and the successful retransformation into the 2H phase by laser processing is indicated by the return of the $E_{2g}^{1}$ and $A_{1g}$ peaks (pink curves), whose intensity even exceeds that of the untreated film. Highest Raman peak intensities are achieved on the ${\mathrm{MoS}}_{2}$ film, which was solely USP laser treated (blue curve). Increased crystallinity after USP laser treatment resulted from an annealing effect and has already been studied on PEALD‐deposited ${\mathrm{MoS}}_{2}$ films [52, 53]. Successful retransformation of lithium‐ion treated ${\mathrm{MoS}}_{2}$ films into the stable 2H‐phase, with improved crystallinity is observed after lithium ion irradiation. The intensity mapping in the laser path is modulated closely approximating the Gaussian intensity profile of the USP laser beam. Areas in the implantation that have been retransformed appear visibly brighter in optical images, indicating that color alterations by ion beam exposure are largely driven by changes in optical parameters rather than thickness variation.

**FIGURE 7:**
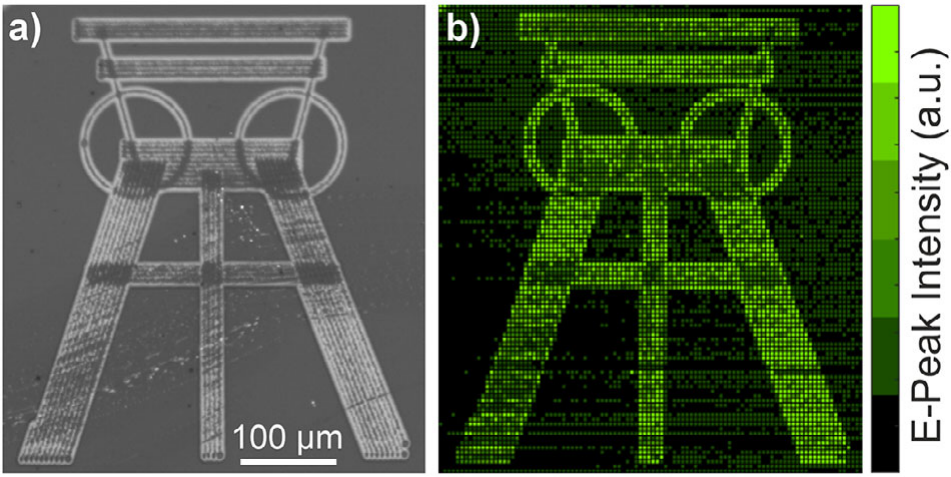
Headframe written by USP laser indicating successful retransformation of ${\mathrm{MoS}}_{2}$ after 1T phase at $D_{9}$ = $4.64\times 10^{15}$ ${\mathrm{cm}}^{-2}$ Ion beam treated ${\mathrm{MoS}}_{2}$ sheets. (a) blue light optical microscopy and (b) E‐peak intensity mapping of the trans‐ and retransformed ${\mathrm{MoS}}_{2}$ film.

The performance of this disruptive technology for controlling the phase in a two‐dimensional (2D) material has been demonstrated by using a USP laser to write a headframe, in a circle that has previously been treated by the FIB ion beam. The optical light microscope image Figure 7a shows the different phases, and Raman mapping confirms the presence of the $A_{1g}$ and $E_{2g}^{1}$ Raman peaks of the 2H phase in the USP laser‐written headframe (Figure 7b). Outside the laser path, it is no longer predominant. The lower intensity of the $A_{1g}$ peak at the intersection points is due to the excessive total energy resulting from repeated writing at these points, which leads to the formation of laser‐induced periodic surface structures (LIPSS). LIPSS formation typically occurs within a fluence range that is higher than that required for annealing, but lower than that required for ablation [53]. Raman mapping of the headframe impressively demonstrates the potential of industrial implementation of this heterophase technology.

## 3 Experimental Section

All samples used in this study used ${\mathrm{MoS}}_{2}$ sheets directly grown by PEALD on ${\mathrm{SiO}}_{2}/\mathrm{Si}$‐Substrates consisting of 200 nm thermally grown ${\mathrm{SiO}}_{2}$ on $p^{+}$‐Silicon. The process was conducted inside of a SENTECH‐Instruments PEALD Reactor with Bis(t‐butylimido)bis‐(dimethylamino)molybdenum as molybdenum precursor and hydrogen sulfide $H_{2}S$ as coreactant. Wafer temperatures were approximated to be about 230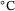. Details of the deposition process were published elsewhere [54]. The ${\mathrm{MoS}}_{2}$‐thickness was measured and mapped on both wafers using a spectroscopic ellipsometer (SENTECH Instruments GmbH, Model SE800‐DUV).

For the ion beam treatment a RAITH Velion FIB‐SEM with a GaBiLi‐Source (gallium, bismuth and lithium) was used. Acceleration voltages of 5 and 35 kV were applied. Dose variations were controlled by varying the beam's dwell time. The aperture diameter is 10 μm for lithium and 20 μm for gallium. For implantations with a 5 kV acceleration voltage, the step size was 61.3 nm, with beam currents of 29.3 and 15.7 pA, respectively, resulting in dwell times from 2.05 to 952 μs (lithium) and 3.84 μs to 1.78 ms (gallium). For implantations with an acceleration voltage of 35 kV, reduced step sizes of 10 nm, currents of 2.9 pA for lithium and 10 pA for gallium were set. Dwell times equate to at 551 ns up to 255 μs for lithium and 160 ns to 74 μs for gallium. The spot‐size at 35 kV acceleration voltage with small currents is about 4 nm for lithium and 6 nm for the gallium ion beam.

Phase transformation using an ultrashort pulse laser was performed with a femtosecond pulsed laser (model Carbide, Light Conversion, Lithuania). The following parameters have been chosen for the experiments: λ=1030 nm, $t_{p}=191$ fs, repetition rate of $f_{rep}=2$ MHz, and an argon atmosphere. The laser fluence f was fixed to 15.82 ${\mathrm{Jcm}}^{-2}$ (equivalent to a pulse energy $E_{P}=50$ nJ) by a half ‐ wavelength (λ/2‐wave plate) and a polarization beam splitter. The laser beam is guided to a galvanometer scanner and focused on the sample surface using a F−Φ lens, with a beam diameter of d≥ 20.6 μm. The phase transition was induced with unidirectional scanning with a scan speed $v_{scan}$ = 20 ${\mathrm{mms}}^{-1}$. Each line was shifted vertically by Δy = 5 μm. This results in a number of spots per size of n=6660 and a percentage pulse overlap in X of 99.95% and in Y of 75.73%. All laser experiments were conducted under Argon atmosphere. The laser fluence f was calculated with the pulse energy $E_{P}$ and the average laser power $P_{L}$:

$$f=\frac{E_{P}}{\pi \omega_{0}^{2}}$$

with

$$E_{P}=\frac{P_{L}}{f_{rep}}$$

These laser parameters were chosen based on a detailed study of the laser process windows for these PEALD ${\mathrm{MoS}}_{2}$ films [24, 55].

Topographical characterization by atomic force microscopy (AFM) was done using the tapping mode on a Nanoscope Multimode V microscope from Digital Instruments. The crystallinity was examined with a Renishaw InVia Raman microscope at P≤15 mW and λ=532 μm. Exposure time was set to 30 s for mappings and 60 s for singular measurements. Graphs of the mappings were created by plotting mean and standard deviation of 8 measurement points each to reduce noise.

HR‐TEM of the irradiated sheets was conducted with a Carl Zeiss Libra 200 MC Cs STEM tool, operating at 200 kV.

Stoichiometric changes in our ${\mathrm{MoS}}_{2}$ samples were characterized by Rutherford Backscattering Spectrometry (RBS), carried out at the 4 MV tandem accelerator of the RUBION facility (Ruhr University Bochum, Germany) using a 2 MeV 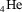 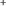 ion beam. The sample was mounted at a 7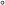 angle to the helium beam and the detector was placed at an angle of 160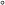. For stoichiometry determination from the RBS spectra, the program SIMNRA [56] was employed.

## 4 Conclusions

The approach presented here for phase trans‐ and retransformation using FIB and USP laser is very promising, as it has been successfully applied to polycrystalline 2D films for the first time and enables extremely high‐resolution control of the phase. The innovative GaBiLi source is an excellent opportunity to create immense added value for 2D electronics due to the low mass of Li ions coupled with the high long‐term stability of the 1T phase under Li intercalation. This source also enables the direct comparison of changes in the properties of ${\mathrm{MoS}}_{2}$ deposited by PEALD under the influence of Ga‐ and Li‐ion beams. The ablation threshold for heavy Ga ions at an acceleration voltage of 35 kV is around $10^{13}$ ${\mathrm{cm}}^{-2}$, whereas for lighter Li ions it is closer to $10^{15}$ ${\mathrm{cm}}^{-2}$. Higher acceleration voltages result in lower ablation thresholds allowing faster ion beam milling process whereas controlled phase transformation should be carried out at lower acceleration voltages. No phase transition was observed for the gallium ions because the films were amorphized immediately within the selected parameter range. In contrast for Li ion doses of $4.64\times 10^{15}$ ${\mathrm{cm}}^{-2}$ at 5 kV acceleration voltage the 2H signal disappears, while the layered structure of the films remains, and only a small amount of excess sulfur was removed by the ion beam indicating the desired phase transformation. The layer properties remained stable for over 19 month.

Retransformation into the 2H phase following irradiation with an Li‐ion beam is successful when USP laser treatments are applied to the ${\mathrm{MoS}}_{2}$ films. As a mask‐free and wafer‐compatible technique, FIB and USP laser processing bridges laboratory‐scale phase engineering and scalable 2D electronics, establishing phase control as a design degree of freedom for next‐generation electronic and optoelectronic systems.

## Conflicts of Interest

The authors declare no conflicts of interest.

## Supporting information


**Supporting File**: smll74834‐sup‐0001‐SuppMat.pdf.

## Data Availability

The data that support the findings of this study are available from the corresponding author upon reasonable request.

## References

[1] R. Mathew and J. Ajayan , “Material Processing, Performance and Reliability of MoS2 Field Effect Transistor (FET) Technology‐ A Critical Review,” Materials Science in Semiconductor Processing 160 (2023): 107397, 10.1016/j.mssp.2023.107397.

[2] K. Kwak , H. Yoon , S. Hong , and B. H. Kang , “Advances in 2D Molybdenum Disulfide Transistors for Flexible and Wearable Electronics,” Micromachines 15, no. 12 (2024), 10.3390/mi15121476. PMC11728206 39770229

[3] R. Thayil , S. R. Parne , and C. V. Ramana , “2D MoS2 for Next‐Generation Electronics and Optoelectronics: From Material Properties to Manufacturing Challenges and Future Prospects,” Small (Weinheim an der Bergstrasse, Germany) 21, no. 14 (2025): e2412467, 10.1002/smll.202412467. 40026204

[4] H. Yang , X. Chen , D. Wu , and X. Fang , “High–Performance Flexible MoS 2 Transistors Using Au/Cr/Al/Au as Source/Drain Electrodes (Adv. Electron. Mater. 12/2023),” Advanced Electronic Materials 9, no. 12 (2023), 10.1002/aelm.202370056.

[5] S. Das , H.‐Y. Chen , A. V. Penumatcha , and J. Appenzeller , “High Performance Multilayer MoS2 Transistors With Scandium Contacts,” Nano Letters 13, no. 1 (2013): 100–105, 10.1021/nl303583v. 23240655

[6] C. Feng , W. Wu , H. Liu , et al., “Emerging Opportunities for 2D Materials in Neuromorphic Computing,” Nanomaterials (Basel, Switzerland) 13, no. 19 (2023), 10.3390/nano13192720. PMC10574516 37836361

[7] J. R. Schaibley , H. Yu , G. Clark , et al., “Valleytronics in 2D Materials,” Nature Reviews Materials 1, no. 11 (2016), 10.1038/natrevmats.2016.55.

[8] J. Kabel , S. Sharma , A. Acharya , D. Zhang , and Y. K. Yap , “Molybdenum Disulfide Quantum Dots: Properties, Synthesis, and Applications,” C 7, no. 2 (2021): 45, 10.3390/c7020045.

[9] S. Michaelis de Vasconcellos , D. Wigger , U. Wurstbauer , A. W. Holleitner , R. Bratschitsch , and T. Kuhn , “Single–Photon Emitters in Layered Van der Waals Materials,” physica status solidi (b) 259, no. 4 (2022), 10.1002/pssb.202100566.

[10] M. Ghosh Dastidar , I. Thekkooden , P. K. Nayak , and V. Praveen Bhallamudi , “Quantum Emitters and Detectors Based on 2D van der Waals Materials,” Nanoscale 14, no. 14 (2022): 5289–5313, 10.1039/D1NR08193D. 35322836

[11] Y. Cao , “Roadmap and Direction Toward High‐Performance MoS2 Hydrogen Evolution Catalysts,” ACS Nano 15, no. 7 (2021): 11014–11039, 10.1021/acsnano.1c01879. 34251805

[12] W. Zhai , Z. Li , Y. Wang , et al., “Phase Engineering of Nanomaterials: Transition Metal Dichalcogenides,” Chemical Reviews 124, no. 7 (2024): 4479–4539, 10.1021/acs.chemrev.3c00931. 38552165

[13] Y. Xiao , M. Zhou , J. Liu , J. Xu , and L. Fu , “Phase Engineering of Two‐Dimensional Transition Metal Dichalcogenides,” Science China Materials 62, no. 6 (2019): 759–775, 10.1007/S40843-018-9398-1.

[14] L. Liu , J. Wu , L. Wu , et al., “Phase‐Selective Synthesis of 1T' MoS2 Monolayers and Heterophase Bilayers,” Nature Materials 17, no. 12 (2018): 1108–1114, 10.1038/S41563-018-0187-1. 30323336

[15] R. Yang , L. Mei , Z. Lin , et al., “Intercalation in 2D Materials and In Situ Studies,” Nature Reviews Chemistry 8, no. 6 (2024): 410–432, 10.1038/S41570-024-00605-2. 38755296

[16] H. Chen , J. Zhang , D. Kan , et al., “The Recent Progress of Two‐Dimensional Transition Metal Dichalcogenides and Their Phase Transition,” Crystals 12, no. 10 (2022): 1381, 10.3390/cryst12101381.

[17] M. A. Py and R. R. Haering , “Structural Destabilization Induced by Lithium Intercalation in MoS2 and Related Compounds,” Canadian Journal of Physics 61, no. 1 (1983): 76–84, 10.1139/p83-013.

[18] S. Das , G. Swain , and K. Parida , “One Step Towards the 1T/2H‐MoS 2 Mixed Phase: A Journey From Synthesis to Application,” Materials Chemistry Frontiers 5, no. 5 (2021): 2143–2172, 10.1039/D0QM00802H.

[19] J. Zhu , Z. Wang , H. Yu , et al., “Argon Plasma Induced Phase Transition in Monolayer MoS2,” Journal of the American Chemical Society 139, no. 30 (2017): 10216–10219, 10.1021/jacs.7b05765. 28731708

[20] J. Byeon , H. Y. Choi , J. Y. Shin , S. R. Jeong , S. K. Gautam , and H.‐D. Lee , “Phase Engineering of MoS2 via Ar/O2 Plasma Treatment,” Materials Letters 410 (2026): 140225, 10.1016/j.matlet.2026.140225.

[21] M. Xiao , Z. Wu , G. Liu , X. Liao , J. Yuan , and Y. Zhou , “Spatially Controlled Phase Transition in MoTe2 Driven by Focused Ion Beam Irradiations,” ACS Applied Materials & Interfaces 16, no. 24 (2024): 31747–31755, 10.1021/acsami.4c03546. 38839057

[22] X. Sun , Z. Wang , and Y. Q. Fu , “Defect‐Mediated Lithium Adsorption and Diffusion on Monolayer Molybdenum Disulfide,” Scientific Reports 5, no. 18712 (2015), 10.1038/srep18712. PMC4686938 26692345

[23] B.‐M. Liu , W.‐P. Xu , X. Long , Y.‐N. Zhang , and J.‐X. Cao , “Role of Rotation Angle and Grain Boundary in Tuning the Li Intercalation Concentration to Induce Phase Transition in Bilayer MoS2,” The Journal of Physical Chemistry C 126, no. 19 (2022): 8539–8544, 10.1021/acs.jpcc.2c01756.

[24] M. Becher , L. Willeke , M. Wilken , A. Devi , C. Bock , and A. Ostendorf , “Ultrashort Pulse Laser Phase Transition of 2D MoS2 Produced by Atomic Layer Deposition,” in LiM 2023 Proceedings (Wissenschaftliche Gesellschaft Lasertechnik und Photonik e.V. (WLT), 2023), 8.

[25] W. Li , X. Zhan , X. Song , et al., “A Review of Recent Applications of Ion Beam Techniques on Nanomaterial Surface Modification: Design of Nanostructures and Energy Harvesting,” Small (Weinheim an der Bergstrasse, Germany) 15, no. 31 (2019): e1901820, 10.1002/smll.201901820. 31166661

[26] K. Höflich , G. Hobler , F. I. Allen , et al., “Roadmap for Focused Ion Beam Technologies,” Applied Physics Reviews 10, no. 4 (2023), 10.1063/5.0162597.

[27] M. Telkhozhayeva and O. Girshevitz , “Roadmap Toward Controlled Ion Beam–Induced Defects in 2D Materials,” Advanced Functional Materials 34, no. 45 (2024), 10.1002/adfm.202404615.

[28] X. Wu , X. Luo , H. Cheng , R. Yang , and X. Chen , “Recent Progresses on Ion Beam Irradiation Induced Structure and Performance Modulation of Two‐Dimensional Materials,” Nanoscale 15, no. 20 (2023): 8925–8947, 10.1039/D3NR01366A. 37102719

[29] B. Tang , Y. Zhao , C. Zhou , et al., “Threshold Voltage Modulation in Monolayer MoS2 Field‐Effect Transistors via Selective Gallium Ion Beam Irradiation,” Science China Materials 65, no. 3 (2022): 741–747, 10.1007/S40843-021-1782-y.

[30] D. Wang , Y. Wang , X. Chen , et al., “Layer‐by‐Layer Thinning of Two‐Dimensional MoS2 Films by Using a Focused Ion Beam,” Nanoscale 8, no. 7 (2016): 4107–4112, 10.1039/C5NR05768J. 26821788

[31] D. S. Fox , Y. Zhou , P. Maguire , et al., “Nanopatterning and Electrical Tuning of MoS2 Layers With a Subnanometer Helium Ion Beam,” Nano Letters 15, no. 8 (2015): 5307–5313, 10.1021/acs.nanolett.5b01673. 26154305

[32] S. W. Han , W. S. Yun , M. Kang , S. Lee , and J. Park , “Phase Transition of a MoS2 Monolayer Through Top Layer Desulfurization by He+ Ion Irradiation,” Journal of Applied Physics 131, no. 22 (2022), 10.1063/5.0092955.

[33] A. Nadzeyka , T. Richter , P. Mazarov , F. Meyer , A. Ost , and L. Bruchhaus , “Focused Ion Beams From GaBiLi Liquid Metal Alloy Ion Sources for Nanofabrication and Ion Imaging,” Journal of Vacuum Science & Technology B 41, no. 6 (2023), 10.1116/6.0002918.

[34] N. Klingner , G. Hlawacek , P. Mazarov , W. Pilz , F. Meyer , and L. Bischoff , “Imaging and Milling Resolution of Light Ion Beams From Helium Ion Microscopy and FIBs Driven by Liquid Metal Alloy Ion Sources,” Beilstein Journal of Nanotechnology 11 (2020): 1742–1749, 10.3762/bjnano.11.156. PMC7684691 33282621

[35] Z. Fekri , P. Chava , G. Hlawacek , et al., “Tuning the Electronic Characteristics of Monolayer MoS 2 –Based Transistors by Ion Irradiation: The Role of the Substrate,” Advanced Electronic Materials 10, no. 9 (2024), 10.1002/aelm.202400037.

[36] L. Dong , J. Yang , X. Yue , S. Dong , W. Li , and X. Li , “Defect Engineering of MoS 2 Nanosheets by Heavy‐Ion Irradiation for Hydrogen Evolution,” ACS Applied Nano Materials 6, no. 20 (2023): 18858–18868, 10.1021/acsanm.3c03165.

[37] X. Wu , X. Zhu , and B. Lei , “Impact of Ion Beam Irradiation on Two‐Dimensional MoS2: A Molecular Dynamics Simulation Study,” Journal of Physics: Condensed Matter 34, no. 5 (2021), 10.1088/1361-648X/ac31f9. 34673551

[38] R. Mupparapu , M. Steinert , A. George , et al., “Facile Resist–Free Nanopatterning of Monolayers of MoS 2 by Focused Ion–Beam Milling,” Advanced Materials Interfaces 7, no. 19 (2020), 10.1002/admi.202000858.

[39] B. K. Bareth , M. Khan , A. Tripathi , and M. N. Tripathi , “Ion‐Irradiation Induced Structural, Electronic, and Optical Properties Modification in a Few Layered MoS2,” Nuclear Instruments and Methods in Physics Research Section B: Beam Interactions with Materials and Atoms 554 (2024): 165436, 10.1016/j.nimb.2024.165436.

[40] X. Liu , D. Zemlyanov , S. Sharma , et al., “Focused Helium Ion Beam for Direct Patterning of Monolayer MoS 2 Nanoribbon Field Effect Devices,” Advanced Functional Materials (2025), 10.1002/adfm.202514880.

[41] F. Mehmood , R. Pachter , T. C. Back , J. J. Boeckl , R. T. Busch , and P. R. Stevenson , “Two‐Dimensional MoS2 2H, 1T, and 1T' Crystalline Phases With Incorporated Adatoms: Theoretical Investigation of Electronic and Optical Properties,” Applied Optics 60, no. 25 (2021): G232–G242, 10.1364/AO.433239. 34613214

[42] Q. Chen , M. A. Gosalvez , Q. Li , and Y. Xing , “Helium Focused Ion Beam Induced Subsurface Damage on Si and SiC Substrates: Experiments and Generative Deep Neural Network Modeling via Position‐Dependent Input,” Journal of Materials Research and Technology 24 (2023): 3363–3382, 10.1016/j.jmrt.2023.03.229.

[43] A. Maddouri , J. R. Durán‐Retamal , S. H. Domingues , et al., “Raman of 2D‐MoS2: Disentangling the Metallic Phase Conundrum,” Small Methods 9, no. 11 (2025): e01129, 10.1002/smtd.202501129. PMC12641350 40899518

[44] J. F. Ziegler , M. D. Ziegler , and J. P. Biersack , “SRIM – The Stopping and Range of Ions in Matter (2010),” Nuclear Instruments and Methods in Physics Research Section B: Beam Interactions with Materials and Atoms 268, no. 11‐12 (2010): 1818–1823, 10.1016/j.nimb.2010.02.091.

[45] M. E. Schmidt , M. Akabori , and H. Mizuta , “Nitrogen Ion Microscopy,” In Ion Beam Applications, eds. I. Ahmad and M. Maaza , (Erscheinungsort nicht ermittelbar: IntechOpen, 2018).

[46] A. V. Krasheninnikov , “Are Two‐Dimensional Materials Radiation Tolerant?,” Nanoscale Horizons 5 (2020): 1447–1452, 10.1039/D0NH00465K. 32969454

[47] J. Bernède , “About the Preferential Sputtering of Chalcogen From Transition Metal Dichalcogenide Compounds and the Determination of Compound Stoichiometry From XPS Peak Positions,” Applied Surface Science 171, no. 1 (2001): 15–20, 10.1016/S0169-4332(00)00535-3.

[48] R. G. Dickinson and L. Pauling , “The Crystal Structure of Molybdenite,” Journal of the American Chemical Society 45, no. 6 (1923): 1466–1471, 10.1021/ja01659a020.

[49] L. Yang , A. Mukhopadhyay , Y. Jiao , et al., “Aligned and Stable Metallic MoS 2 on Plasma‐Treated Mass Transfer Channels for the Hydrogen Evolution Reaction,” Journal of Materials Chemistry A 5, no. 48 (2017): 25359–25367, 10.1039/C7TA08400E.

[50] Y. Zhang , Z. Mu , C. Yang , et al., “Rational Design of MXene/1T–2H MoS 2 –C Nanohybrids for High–Performance Lithium–Sulfur Batteries,” Advanced Functional Materials 28, no. 38 (2018), 10.1002/adfm.201707578.

[51] G. Eda , H. Yamaguchi , D. Voiry , T. Fujita , M. Chen , and M. Chhowalla , “Photoluminescence From Chemically Exfoliated MoS2,” Nano Letters 11, no. 12 (2011): 5111–5116, 10.1021/nl201874w. 22035145

[52] M. Becher , L. Willeke , C. Bock , and A. Ostendorf , “Post Treatment of Plasma Enhanced Atomic Layer Deposited MoS_2_ via Ultra Short Pulse Laser to Increase the Crystallinity,” in Laser + Photonics for Advanced Manufacturing, (SPIE, 2024), 13005, 10.1117/12.3016372.

[53] M. J. M. J. Becher , J. Jagosz , R.‐M. Neubieser , et al., “Ultrashort–Pulsed–Laser Annealing of Amorphous Atomic–Layer–Deposited MoS 2 Films,” Advanced Engineering Materials 25, no. 21 (2023), 10.1002/adem.202300677.

[54] J. Jagosz , L. Willeke , N. Gerke , et al., “Wafer‐Scale Demonstration of Polycrystalline MoS2 Growth on 200 mm Glass and SiO2/Si Substrates by Plasma‐Enhanced Atomic Layer Deposition,” Advanced Materials Technologies n/a, no. n/a (2024): 2400492, 10.1002/admt.202400492.

[55] M. J. M. J. Becher , J. B. Ullrich , J. Jagosz , et al., “Pulse Duration Dependent Formation of Laser‐Induced Periodic Surface Structures in Atomic Layer Deposited MoS2,” Advanced Materials Interfaces 11, no. 35 (2024): 2400478, 10.1002/admi.202400478.

[56] Matej Mayer , “SIMNRA User's Guide. Report IPP 9/113,” (1997).

