# Supplementary material for: Phase Engineering of Plasma‐Enhanced Atomic Layer Deposited Molybdenum Disulfide Using High Accuracy Direct Writing With Focused Li+ and Ga+ Ion Beams and Ultrashort Pulse Laser
Source: Small. 2026 Jul 25;22(53):e74834. doi: 10.1002/smll.74834 (PMC13592421; doi:10.1002/smll.74834)
Supplement: Supplementary file 1 [file SMLL-22-e74834-s001.pdf]

Phase engineering of plasma-enhanced atomic layer deposited molybdenum disulfide using high accuracy direct writing with focused Li^+ and Ga^+ ion beams and ultrashort pulse laser

*Marvin Krüner Achim Nadzeyka Malte Jonas Marius Julian Becher Zhongquan Liao Detlef Rogalla André Clausner Andreas Ostendorf Michael Kahl Claudia Bock**

M. Krüner, C. Bock
Microsystems Technology Ruhr University Bochum Universitätsstr. 150, 44801 Bochum, Germany
Email Address: claudia.bock@rub.de
A. Nadzeyka, M. Kahl
RAITH GmbH, Konrad-Adenauer-Allee 8, 44263 Dortmund, Germany
Z. Liao, A. Clausner
Fraunhofer Institute for Ceramic Technologies and Systems IKTS, Maria-Reiche-Str. 2 01109 Dresden, Germany
D. Rogalla
RUBION, Ruhr University Bochum Universitätsstr. 150, 44801 Bochum, Germany
M. J. M. J. Becher, A. Ostendorf
Applied Laser Technologies, Ruhr University Bochum Universitätsstr. 150, 44801 Bochum, Germany

Keywords: *phase engineering, intercalation, focussed ion beam, MoS_2, ultrashort pulse laser, PEALD*

1 Test design for FIB implantations

The set of implantations was done on a sample of approximately 10 nm thick MoS_2 on 200 nm thermally grown SiO_2 on silicon. To accurately align the FIB-SEM system we designed a layout with alignment marks to define a square 120 µm writing field. Following UV-lithography we used aluminum evaporation and a liftoff in acetone to create high contrast alignment marks. Afterwards we implanted an array of 20 µm × 20 µm squares with gallium and lithium each as can be seen in Figure S1 a. The implantation dose increased exponentially from one square to the next (see Figure S1 d), while the acceleration voltage remained fixed at 5 kV and 35 kV respectively, creating a controlled dose gradient for comparison.

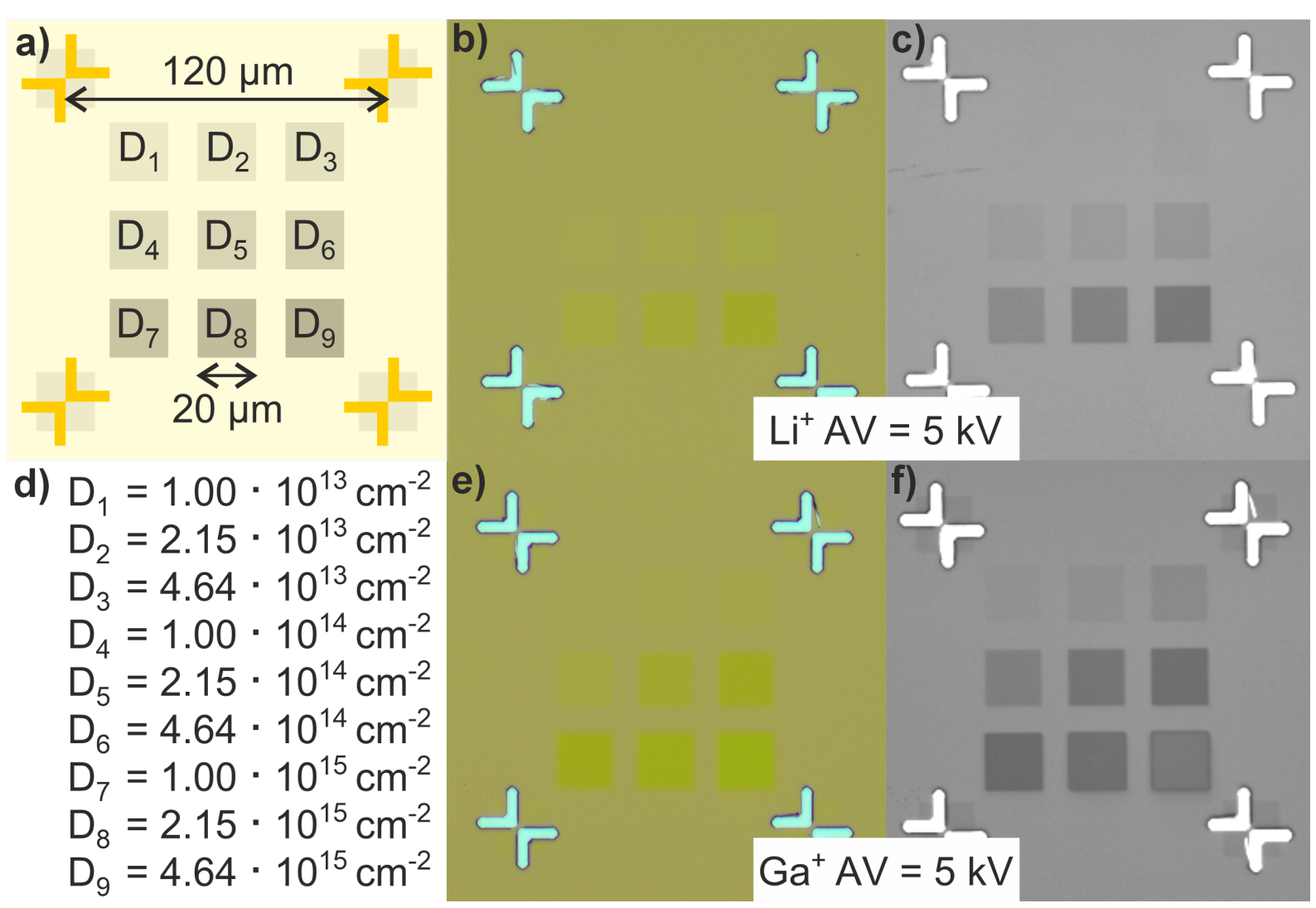

Figure S 1: a), d) Schematic depiction of the lateral setup of process marks and implantations used for the first results with a list of implanted ion dosages. b), e) optical microscopy images of Lithium and Gallium implantations in a writing field with acceleration voltages of 5 kV. c), f) blue light optical microscopy images of the same implantations.

Optical micrography with white and blue illumination (Figure S1 b, c and e, f respectively) clearly display changes in color and contrast with rising dosages. Color differences are more pronounced after gallium ion treatment due to increased sheet ablation and amorphization of the film.

2 Raman analysis

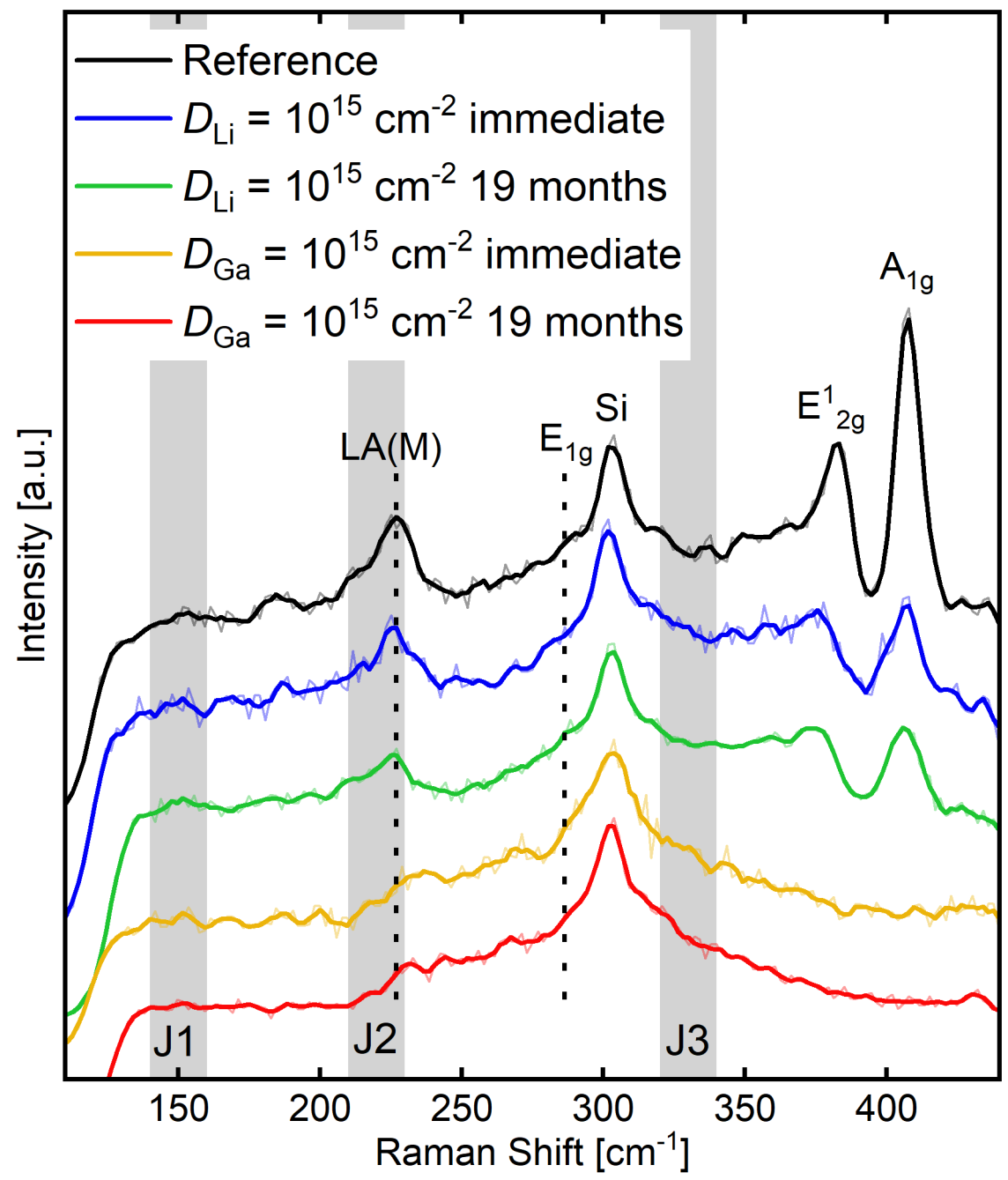

Figure S 2: Raman Spectra of MoS_2 irradiated with $10^{15}cm^{-2}$ ion doses of 5 keV lithium and gallium ions immediately after implantation and 19 months later compared to a unirradiated areas. Each graph consists of one half transparent graph depicting the original data and a non transparent line showing the data Savitzky-Golay filtered with a window of 6 data points to reduce noise.

Figure S2 shows Raman spectra of comparable lithium and gallium implantations directly after implantation and 19 months later and a reference measurement in an extended range from 110 cm^{-1} to 440 cm^{-1}. The relevant peaks as well as the potential peak positions of J_1, J_2, J_3 and E_{1g} are indicated. The E_{1g} mode is symmetry-forbidden in backscattering geometry and, for thin films on Si, is further obscured by the Si substrate band. We observe E_{1g} as a shoulder within the broad Si peak of our reference and lithium ion beam treated samples since our polycrystalline layer contains out-of-plane crystallites [1].
In our setup, the low-wavenumber edge filter cuts off close to the expected J_1 position, placing J_1 at the detection limit. Moreover, Maddouri et al. demonstrated that features in the J_1 region can originate from molybdenum oxides or substoichiometric MoO_x, implying possible misassignments in disordered or partially oxidized samples [2]. For this reason, the observation of the J_2 peak near 222 cm^{-1} would constitute strong evidence for the 1T phase.
In practice, J_2 is rarely detected even in monocrystalline MoS_2 and, to our knowledge, has not been observed in polycrystalline MoS_2 because the expected J_2 feature overlaps with the disorder-activated LA(M) band, precluding reliable deconvolution of separate components. We therefore performed a Lorentzian fit after normalization to the Si peak, using the 15 data points closest to the combined $LA(M)$/putative J_2 feature in the reference and Li-irradiated spectra at a dose of $10^{15}cm^{-2}$. The fitted peak positions are 226.3 cm^{-1} (reference), 225.3 cm^{-1} (immediately after Li implantation), and 225.5 cm^{-1} (after 19 months). The long time stable shift toward the expected J_2 position, away from the $LA(M)$ band, supports a successful phase transition.
To the best of our knowledge, distinct J-peaks have not been reported for MoS_2 grown by plasma-enhanced atomic layer deposition (PEALD); even PEALD films converted by wet-chemical intercalation do not exhibit J-peaks. Consequently, distinct J features are not expected here.

3 Damage to crystalline Si caused by Li^+ ions

Lithium ion beams at 35 keV deteriorates the silicon Raman peak intensity at 521 cm^{-1}. Figure S3 show Raman spectra of MoS_2 films treated with ion doses from 0 to $4.64 \cdot 10^{15} cm^{-2}$ at 35 kV acceleration voltage. The Silicon Peaks intensity at 521 cm^{-1} is visibly reduced with rising ion dose. This is attributable to severe substrate damage by the high energy lithium ions. Simulation results in the main text (Figure 5) show a clear penetration of the Silicon substrate with lithium at this high ion energy.

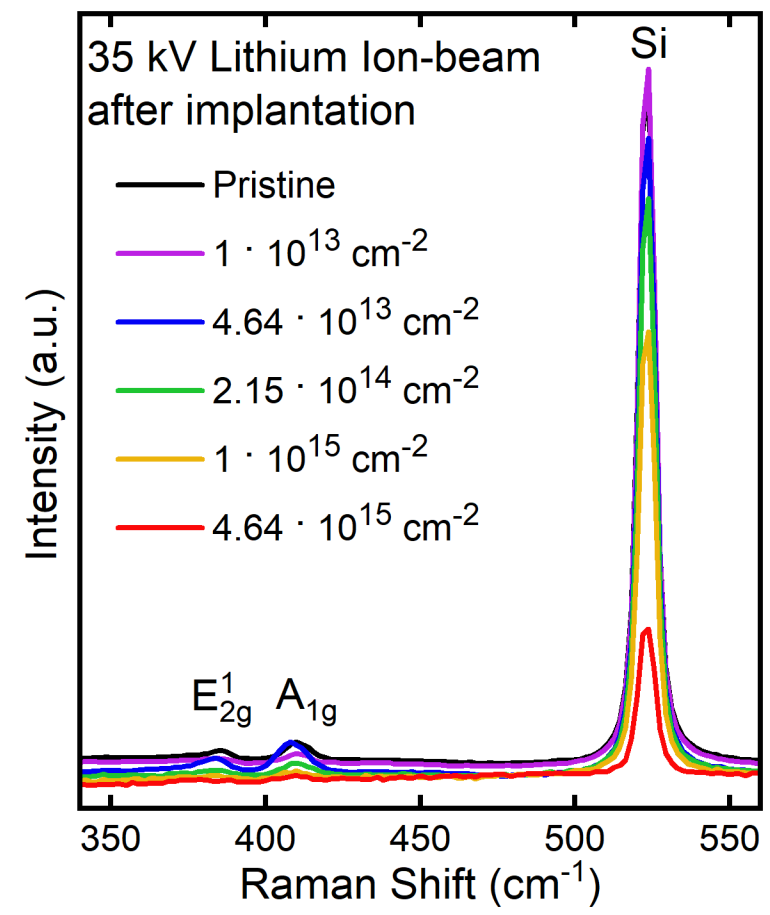

Figure S 3: Raman Spectra of MoS_2 irradiated with increasing ion doses of 35 keV Lithium ions compared to a unirradiated areas.

4 Large area ion implantation

Circular implantations with a diameter of 750 µm were implanted with two different gallium and one lithium dose. These were later used for Rutherford backscattering spectrometry whose results can be found in the main text. Raman spectroscopy and optical images with red, blue and green light sources can be seen in Figure S4.

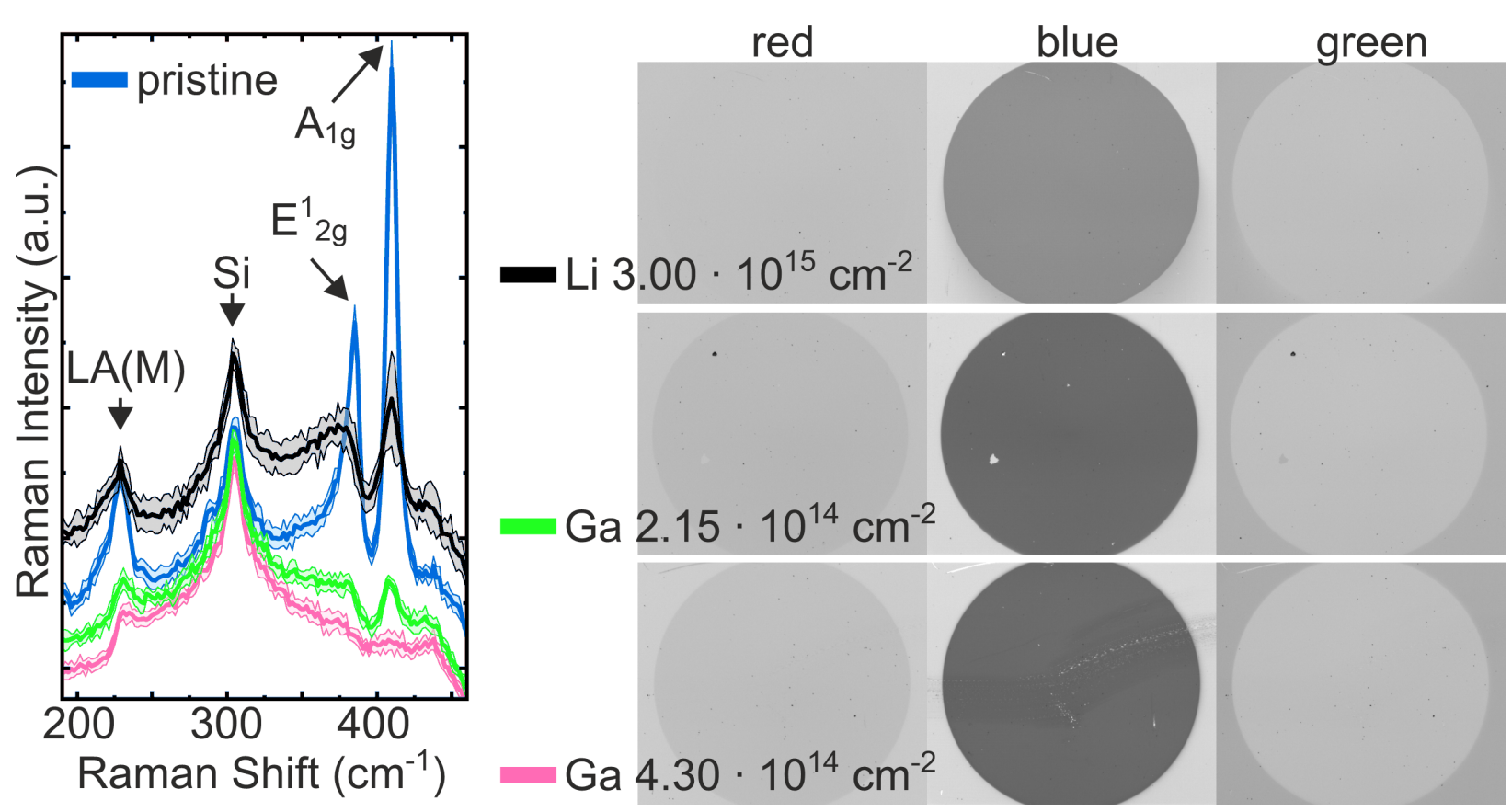

Figure S 4: Raman spectra of a Pristine MoS_2-layer compared to FIB irradiated MoS_2 (left). Grey-scale optical microscopy images with different color illumination of the circular FIB irradiated MoS_2 samples.

5 Grid patterns for reliable determination of ablation

To more accurately measure the ablation by ion beam and compare our results with the literature we implanted a grid structure using lithium and gallium at both 5 kV and 35 kV acceleration voltage. The

layer thickness that was removed can be found in figure 4 (main text). Optical microscopy images of said implantations can be seen in Figure S5. The grids middle rectangle is 10 µm wide and both left and right rectangles 5 µm. Dosage was varied from 10^{13} cm^{-2} (upper left implantation) to 10^{16} cm^{-2} (lower right implantation).

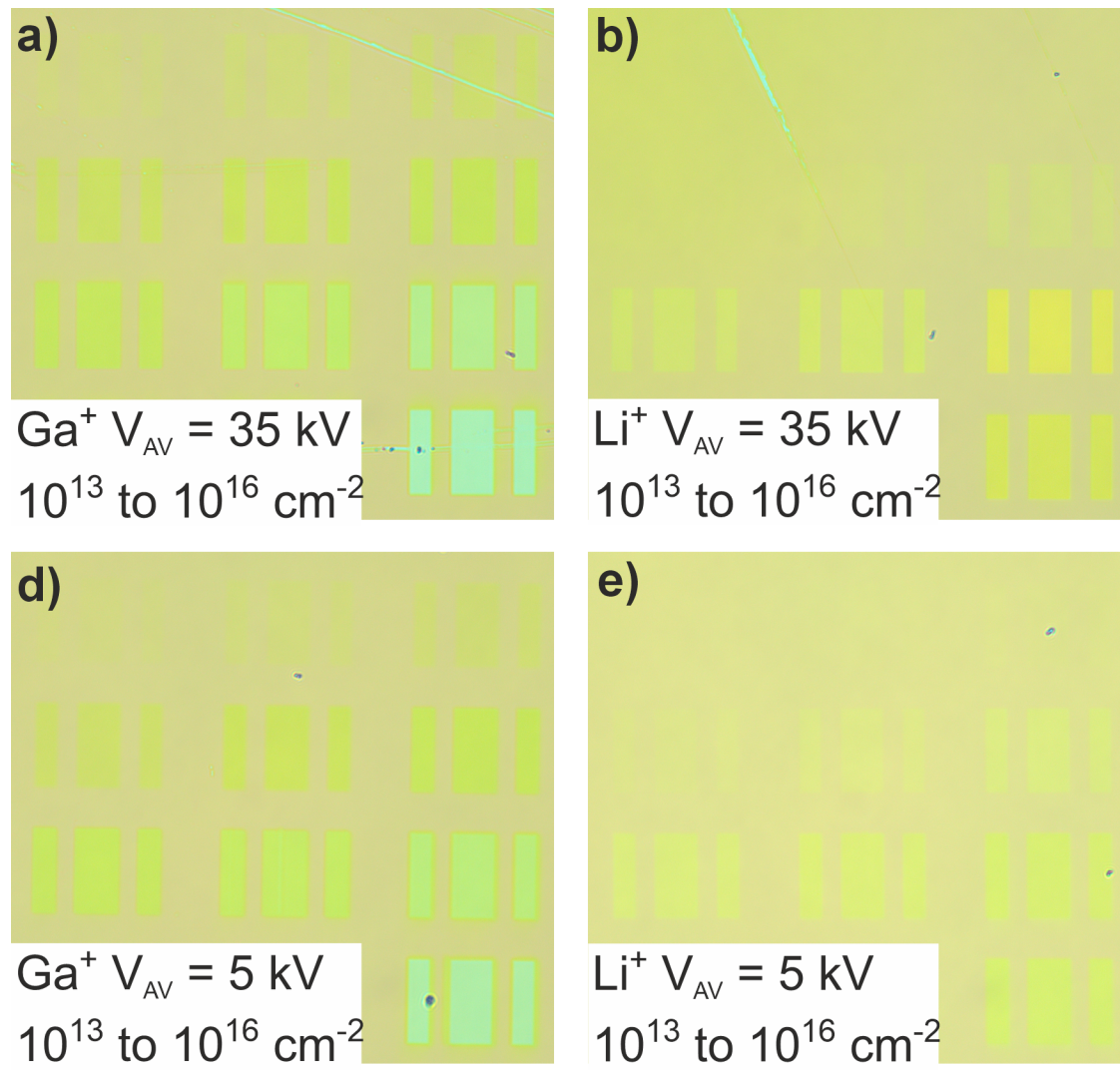

Figure S 5: Optical microscopy images of implanted grid structures on MoS_2 done by Gallium and lithium ion irradiation.

References

[1] É. Blanco, P. Afanasiev, G. Berhault, D. Uzio, S. Loridant, *Comptes Rendus. Chimie* **2016**, *19*, 10 1310.

[2] A. Maddouri, J. R. Durán-Retamal, S. H. Domingues, D. L. Rauret, A. Garzón-Manjón, J. Arbiol, O. Ghodbane, F. Touati, R. M. González-Gil, L. N. Bengoa, S. Kouass, P. Gómez-Romero, *Small methods* **2025**, *9*, 11 e01129.
